# Microencapsulation of Flavors in Carnauba Wax

**DOI:** 10.3390/s100100901

**Published:** 2010-01-25

**Authors:** Jelena Milanovic, Verica Manojlovic, Steva Levic, Nevenka Rajic, Viktor Nedovic, Branko Bugarski

**Affiliations:** 1 Faculty of Technology and Metallurgy, University of Belgrade, Karnegijeva 4, 11120 Belgrade, Serbia; E-Mails: millanovic_89@yahoo.com (J.M.); vmanojlovic@tmf.bg.ac.rs (V.M.); nena@tmf.bg.ac.rs (N.R.); 2 Department of Food Technology and Biochemistry, Faculty of Agriculture, University of Belgrade, Nemanjina 6, 11081 Belgrade-Zemun, Serbia; E-Mails: stevalevic@gmail.com (S.L.); vnedovic@agrif.bg.ac.rs (V.N.)

**Keywords:** microencapsulation, carnauba wax, flavors, ethyl vanillin, TG-DSC

## Abstract

The subject of this study is the development of flavor wax formulations aimed for food and feed products. The melt dispersion technique was applied for the encapsulation of ethyl vanillin in wax microcapsules. The surface morphology of microparticles was investigated using scanning electron microscope (SEM), while the loading content was determined by HPLC measurements. This study shows that the decomposition process under heating proceeds in several steps: vanilla evaporation occurs at around 200 °C, while matrix degradation starts at 250 °C and progresses with maxima at around 360, 440 and 520 °C. The results indicate that carnauba wax is an attractive material for use as a matrix for encapsulation of flavours in order to improve their functionality and stability in products.

## 1. Introduction

Flavours are considered as valuable ingredients in any food formula. They are usually expensive, delicate and volatile. Therefore, food manufacturers are usually concerned about the preservation of aromatic additives. Encapsulation offers an effective approach to cover an active compound with a protective wall material and to impart some degree of protection against evaporation, chemical reactions (such as flavour-flavour interactions, light-induced reactions, oxidation) or migration in a food [1,2]. Encapsulation can be employed to retain aroma in food product during processing or storage and/or allow a controlled release.

Different materials have been used to achieve the above application, including proteins, carbohydrates, lipids, fats and gums [1]. Natural waxes are solids which seem suitable for aroma encapsulation; they can provide superior ease of handling, especially of those aromas which are liquid oils, by converting them into powders; they are stable, inert, and considered as safe; they provide a long-term retention of compounds not only with high partition coefficients (*k_o/w_*) (for lipophilic compounds), but also with low ones [3]. Also, natural waxes are of food grade purity, that is, insect waxes like bees wax and plant waxes like candelilla wax and carnauba wax are permitted additives in the European Union (E901-903). They exhibit interesting rheology and microstructure. At room temperature wax is ductile without giving cracks [4–6]. There are indications that in waxes plate-like crystals are formed which are more efficient in hampering the diffusion of small molecule (*i.e.*, low molecular weight) compounds [4,5,7].

Carnauba wax is the hardest, highest-melting, natural commercial wax. It is a plant exudate from the Brazilian ‘tree of life’ (*Copernica cerifera*), composed almost entirely of esters of C24 and C28 carboxylic acids and C32 and C34 straight-chained primary alcohols. Compared to other waxes (such as beeswax), camauba wax is significantly less viscous (and thus easier to manipulate during capsule processing), more elastic, and more resistant to deformations [8]. In foods, it is used as a formulation aid, lubricant, release agent, anticaking agent, and surface finishing agent in baked foods and mixes, chewing gum, confections, frostings, fresh fruits and juices, gravies, sauces, processed fruits and juices, soft candies. Waxes have also been used extensively as carriers for various types of drugs in pharmaceutical applications.

In this study ethyl vanillin (3-ethoxy-4-hydroxybenzaldehyde) was used as the aroma agent. It is used as a substitute for vanilla (3-methoxy-4-hydroxy-benzaldehyde) in foods and perfumes, because it is cheaper and possesses better storage and transport characteristics. Ethyl vanillin is an important food additive as a flavour enhancer. This compound is widely used to contribute to the fragrance of commercial foods such as candies, cookies, chocolate and beverages. However, ethyl vanillin must be added carefully. Large amounts of this flavour cause headaches, nausea and vomiting [9]. In order to determine the smallest amount of the flavour which would give an optimal release profile, the behavior of the encapsulated flavour under heating conditions which mimic these of usual food processing was investigated by thermogravimetric techniques, TG analyses.

Different techniques like spray drying, spray-bed-drying, fluid-bed coating, spray-chilling, spray-cooling or melt injection are available to encapsulate flavors like ethyl vanillin [10,11]. Spray drying is particularly used for encapsulating flavors at the industrial level [10]. Various materials have been used for the encapsulation of ethyl vanillin by this technique, such as gum arabic and modified starches (for example the oxidized starches prepared from corn and waxy amaranth starch) [12]. Starches of different origin have been investigated, with the addition of polysaccharide binding agents (gum arabic, carboxymethylcellulose and carrageenan). In those studies, ethyl vanillin was added at 5 and 10 weight%, based on starch. The best entrapment of ethyl vanillin is achieved with the addition of gum arabic [13]. The spray drying technique is also applied to produce complexes of ethyl vanillin with *β*-cyclodextrin (*β*-CD) [14]. The CDs act as flavour carriers, while furthermore they protect against oxidation, light-induced decomposition, and heat-induced changes. Moreover, CDs show improved shelf life of food products and they mask or reduce undesired smells or tastes, but these hydrophilic materials have little affinity for hydrophobic oils. By modifying them with *n*-octenylsuccinic anhydride it is possible to alter their hydrophilic nature [15]. Although spray-dryers are ubiquitous in the industrial sector, there are a few disadvantages of this technique like complexity of the equipment and nonuniform conditions in the drying chamber; in addition it is often difficult to control particle size. Another technique recently used to encapsulate ethyl vanillin in alginate gel microbeads is electrostatic extrusion technique which is based on the use of electrostatic forces [16]. The obtained results have shown that up to 230 °C most of the encapsulated ethyl vanillin remained intact. Herein we applied the melt dispersion technique to encapsulate ethyl vanillin and thus fulfilled the criterion (considered as one of crucial importance by food manufacturers) of a cheap preparation procedure. This is also the simplest means of encapsulation based on emulsifying the ingredient of interest in a hot solution containing the so-called “wall” material. In comparison to other encapsulation techniques, the production equipment is relatively simple and easy to scale-up.

## 2. Results and Discussion

In an oil-in-water system, such as the one used in this study, the role of an emulsifier, *i.e.*, a wetting agent, is to reduce the surface tension of an oil phase allowing easier spreading and also to lower the interfacial tension between phases. This enables formation of a stable microemulsion. One must select a surfactant or surfactant pair between the huge numbers of available chemical types, with the correct solubility for the unique application. In this study, the mixture of Tween 20 and Span 40 was used as a surfactant agent. Tween surfactants are ethoxylated sorbitan esters while Span surfactants are fatty acid esters of sorbitol. Our selection is based on the chemical similarities between the surfactants and carnauba wax. That is, carnauba wax also consists of esters of hydroxylated unsaturated long-chain fatty acids with long-chain alcohols. This particular blend (the weight ratio Tween 20 to Span 40 is 0.53:0.47) has a hydrophilic-lipophilic balance (HLB) value matching the required hydrophilic-lipophilic balance value (HLB_req_ 12 of carnauba wax).

**Figure 1.**
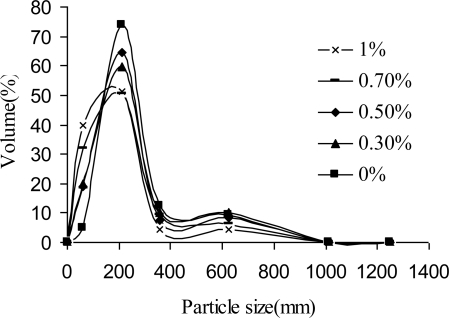
Effect of the surfactant content on the size of distribution of the wax microparticles containing approx. 10% w/w ethyl vanillin.

In Figure 1 the size distribution of the microparticles as a function of the content of the emulsifier is shown. In all cases the size distribution is bi-modal, with the main fraction in the range 210–360 μm and a small second shoulder representing large microparticles of sizes above 500 μm. When the surfactant content was reduced from 1% down to 0% w/w, the distribution slightly narrowed as the prevailing fraction increased from 50 to 74%. This result suggests that under conditions of moderate mixing speed it is possible to make spherical micro sized beads without the addition of any surfactant. The fact that with very few consumables by a simple preparation procedure it is possible to produce micro sized particles makes these wax aroma formulations cost effective and therefore attractive to be used as food supplements in animal feed supply. However, in order to obtain small, uniform and spherical particles which would not adversely affect the overall mouth feel or sensory properties of a food product in humans, it is necessary to optimize a method for the manufacture of wax microparticles. By literature it is estimated that the maximum allowable size for encapsulates in spreads is ∼40 μm, while the smallest solid particles that can be sensed are 22 μm [11,17]. It is believed that this technique could be optimized giving particle sizes well below the in-mouth detection limit or giving softer particles. Especially by introducing higher stirrer speed, the average size may be reduced.

The thermal behavior of wax beads encapsulating ethyl vanillin was investigated by thermogravimetric (TG) and differential scanning calorimetry measurements (DSC) under heating conditions which mimicked usual food processing to provide information about the thermal decomposition of the wax matrix and the kinetics of aroma release. The TG-DSC measurements provide data regarding the melting point, thermal stability, weight loss during heating, as well as data, which can serve for further study of decomposition kinetics. The results could be useful for the formulation studies of food additives as well as for subsequent development of a stable and effective dosage form.

**Figure 2.**
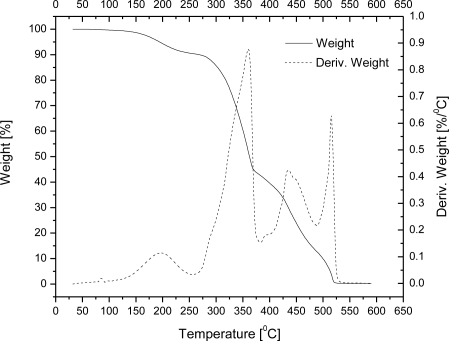
TG curves of wax microparticles entrapping approx. 10% w/w ethyl vanillin.

**Figure 3.**
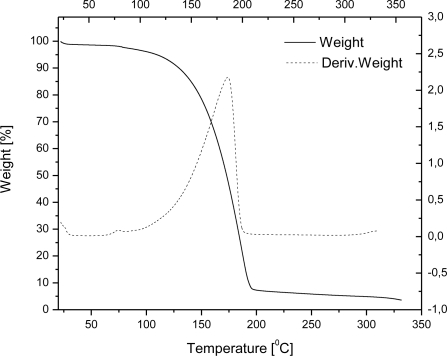
TG and DTG curves of ethyl vanillin.

The TG curves of wax microparticles entrapping ethyl vanillin are shown in Figure 2. Although microparticles have to resist the typical temperatures of baking processes (200–250 °C) in food industry and food preparation applications, we investigated thermal behavior up to ∼550 °C since carnauba wax undergoes decomposition near this temperature. From 20 to 200 °C, microcapsules of wax encapsulating ethyl vanillin exhibited a weight loss of about 6%. The weight loss in this heating range can be reasonably attributed solely to the ethyl vanillin, since carnauba wax starts to evaporate at a far higher temperature (about 250 °C). The presence of ethyl vanillin is also confirmed by the peak in DTG curve at around 198 °C. For a comparison, free ethyl vanillin starts to evaporate at 109 °C (weight loss of 5%) and weight loss occurs at around 186 °C upon heating under same conditions (Figure 3). There are three DTG maxima in temperature range from 200 to 550 °C indicating that weight losses of carnauba wax occur at around 360, 440 and 520 °C (Figure 2). The result strongly supports the conclusion that ethyl vanillin is encapsulated inside the wax matrix and that the encapsulation enables complete retention of the flavour agent up to 200 °C, which is usually the final temperature of baking processes.

**Figure 4.**
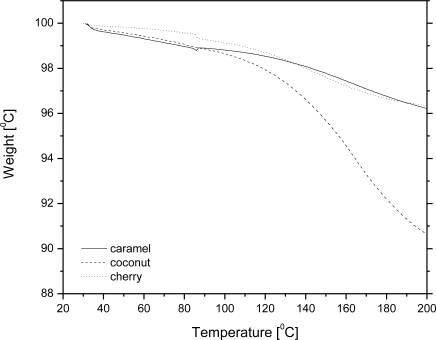
TG curves of wax microparticles entrapping approx. 10% w/w flavour compound.

We also investigated the possibility of using wax to encapsulate several other commercial flavour agents. Figure 4 shows TG curves for wax-flavour microcapsule formulations containing 10% w/w of a flavour (caramel, coconut or cherry). The release kinetics of a flavour compound from wax microbeads depends on the nature itself of a compound, *i.e.*, on interactions between the carrier and a compound. For example, the flavour loss of caramel and cherry was 30% of the initial mass while heating up to 200 °C, while under the same conditions coconut mass loss was almost 100%. Our intent is to show that each system should be analyzed even at a molecular level to get insights into the binding mechanism of wax and an aromatic substance in order to predict the release kinetics of an particular flavouring agent. Flavour agents are usually complex mixtures of different chemical compounds; therefore, prediction of the ability to retain flavours by encapsulating them into the wax carrier would require a detailed insight into their chemical composition. Based on this knowledge, and taking into account that in carbohydrate carrier materials, such is carnauba wax, the order of retention is alcohols > ketones = esters > acids [11], one may predict the release rate; however, one has also to bear in mind that many exceptions to this order have been found. Not only the presence of chemical groups influences the retention or binding, but also other characteristics like polarity, and electroelasticity may play a significant role as well [18].

**Figure 5.**
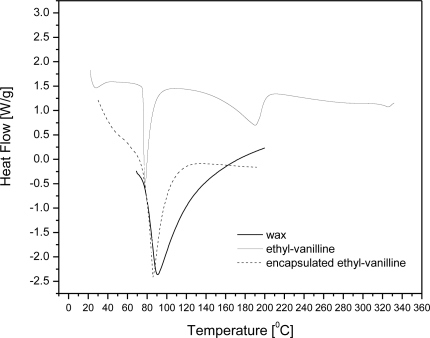
DSC scans of carnauba wax, ethyl vanillin, and wax microcapsules containing ∼10% w/w ethyl vanillin.

Differential scanning calorimetry measurements (DSC) scans of carnauba wax, ethyl vanillin, and wax microcapsules containing ∼10% w/w ethyl vanillin are shown in Figure 5. The thermogram of ethyl vanillin shows a sharp endothermic peak at 78 °C, in agreement with melting point of this substance and a weak one at 190 °C which is attributed to vaporization. However, it is observed that the microcapsule formulation shows no endotherms corresponding to the melting point of ethyl vanillin. This suggests that ethyl vanillin dissolved in the wax matrix as the temperature was raised up to its melting point during the DSC run. From the scans it is also observed that untreated wax and microcapsule formulations show endothermic melting peaks at 90 °C and 86 °C, respectively. According to the DSC scans, carnauba wax has a wide melting temperature range and thermal transition onset is 73 °C. This means that ethyl vanillin and wax melt simultaneously in a certain temperature range.

The slight variations in the enthalpy values and position of the melting point between samples may be explained by polymorphic transitions in the melt-cooled waxes, which are well documented in the literature [19]. However, DSC studies cannot be used to conclusively determine the physical state of ethyl vanillin in wax matrices.

**Figure 6.**
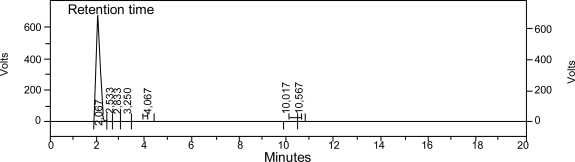
HPLC chromatogram of a sample obtained by extraction of microcapsule compounds in ethanol.

Figure 6 shows a HPLC chromatogram of microparticle compounds extracted in ethanol demonstrating that the microcapsules contain ethyl vanillin. Examination of the UV signal reveals a large peak at 2.083 min, identified as the ethyl vanillin peak. The area under the peak showed that weight of ethyl vanillin is 8.65 ± 0.56% of the total microcapsule weight. The encapsulation efficiency of ethyl vanillin in the microspheres is thus calculated with respect to the total initial input (10% w/w) as 86.5 ± 5.6% w/w.

**Figure 7.**
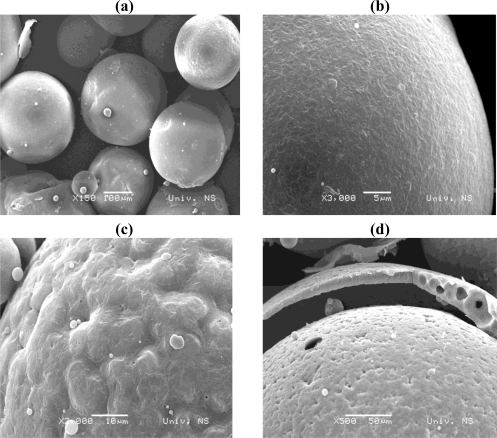
SEM images of wax microcapsules encapsulating 10% w/w ethyl vanillin: (a) with low magnification; (b) with high magnification showing smooth surface; (c) with high magnification showing rough surface; (d) the cracked microcapsule.

Figure 7 shows the SEM photos of the wax microcapsules encapsulating 10% w/w ethyl vanillin. SEM with a low magnification (Figure 7a) shows well separated microparticles with a regular spherical shape. SEM at higher magnification (Figure 7b) reveals that the surface of microparticles is mainly smooth. However, surface irregularities are also observed on some particles, which then appear as rather rough surface capsules (Figure 7c). The fine core/shell microstructure of microcapsules can also be confirmed by the SEM image of a broken microcapsule as shown in Figure 7d. The fracture of surface opened the empty interior of beads and showed a wall thickness of the order of several tenths of micrometers.

## 3. Experimental

### 3.1. Materials

Feed grade carnauba wax was purchased from Carl Roth GmbH (Germany), emulsifiers (Tween 20, Span 40, Span 60) were supplied by Sigma Aldrich (Germany), ethyl vanillin and other aromatic compounds were obtained as a kind gift from Ireks Aroma (Croatia) and Aroma (Serbia).

### 3.2. Preparation of the microparticles

The microparticles entrapping ethyl vanillin were produced by the method which is basically described in the literature [20–24]. Carnauba wax (8% w/w) was melted in purified water containing an emulsifier (a Tween 20/Span 40 mixture in weight ratio 0.53:0.47) at 95 °C in a thermostatted water bath. The content of the emulsifier varied in the range 0–1% w/w. Ethyl vanillin was added to the dispersion of molten wax in water in a 1:10 weight ratio to wax while stirring rigorously (1,200 rpm for 4 min) by a mechanical stirrer with a two blade impeller. The solidification of the micro droplets were performed by adding cold water (2–5 °C) to the resulting dispersion in order to cool it down. Finally, the microparticles were collected by filtration under reduced pressure, washed with water and dried at elevated temperature (50 °C). Volumetric size distributions (volume of microspheres in each diameter class) were determined using a Malvern Mastersizer 2000 (Malvern UK) laser diffraction instrument with a Scirocco 2000 set-up.

### 3.3. HPLC measurements

A Knauer HPLC system equipped with a Knauer UV detector seat at 280 nm and a Supelcosil LC-18-OB (200 mm × 4.6 mm, 5 μm) analytical column was used for ethyl vanillin quantification, using the protocol described in the literature [25–27]. A binary eluent consisting of methanol (A) with 0.5% acetic acid (B) was used. Flow rate was set to 0.8 mL·min^−1^. Elution of the components was achieved by a linear gradient, starting with 0% B and reaching 40% B after 10 min and 100% B after 10 min more. The sample for HPLC analyses was prepared by ethanol extraction of the ethyl vanillin from the microparticles. In short, wax microbeads (200 mg) were placed in concentrated ethanol (45 mL) and exposed to vigorous mixing with an Ultra-Turrax (24,000 rpm) to mechanically disrupt them and allow for extraction; after filtration, the filtrate was collected and analysed using the HPLC method. Standard solution was prepared by dissolving 200 mg of ethyl vanillin in 25 mL of ethanol. The experiment was preformed in triplicate.

### 3.4. Determination of encapsulation efficiency

Encapsulation efficiency is defined as the total amount of encapsulated ethyl vanillin (*m_E_*), divided by the total initial input of ethyl vanillin (*m_I_* = 10% w/w), as given by the following equation:

(1) $$\mathrm{EE}(\%)=\frac{m_{E}}{m_{I}}$$

The total amount of encapsulated ethyl vanillin (*m_E_*) was calculated according from the following formula:

(2) $$m_{E}=c_{M}\times m_{M}$$

where *c_M_* represents the concentration of ethyl vanillin in a solution obtained by extraction of a microcapsules sample in ethanol, and *m_M_* is the weight of microcapsules sample. The concentration of the ethyl vanillin extract *c_M_* was determined by HPLC measurements. This value was calculated by solving a simple proportion:

(3) $$c_{M}=\frac{P_{M}\times c_{S}}{P_{S}}$$

where *P_M_* is the area under the peak of ethyl vanillin in the HPLC chromatogram of a sample solution, *Cs* is the concentration of ethyl vanillin standard solution and *Ps* is the area under the peak of ethyl vanillin in the HPLC chromatogram of ethyl vanillin standard solution. Concentrations of ethyl vanillin in standard and sample solutions are expressed in mg/100 mL.

### 3.5. Thermal analysis

The thermal behavior of the particles was investigated employing the simultaneous DSC-TGA technique using a TA Instruments model SDT Q-600 (New Castle, Delaware, US). The samples (mass approx. 10 mg) were heated in a standard alumina sample pan. All experiments were performed out under dynamic air of a flow rate of 0.1 dm^3^/min using a heating rate of 10 °C/min run.

### 3.6. Scanning electron microscopy

The surface morphology of the microcapsules was imaged using a scanning electron microscopy (SEM). Samples were coated with Au using a Spater coater device Baltec SCD 005. The micrographs are obtained using a SEM-Jeol JSM 6460LV instrument.

## 4. Conclusions

Carnauba wax was used to encapsulate aroma compounds in order to improve aroma functionality and provide protection against evaporation of those volatile actives during processing. We produced spherical micro sized beads with bi-modal size distribution; the main fraction had diameter in the range 210–360 μm. The SEM images revealed the smooth surface and hollow-core/shell microstructure of the microcapsules. The achieved encapsulation efficiency calculated with respect to the total initial input is about 87%. Ethyl vanillin was used as the model flavour compound to investigate the release kinetics by the TG-DSC method. This study show that the decomposition process under heating proceeds in several steps: vanillin evaporation occurs at around 200 °C, while matrix degradation starts at 250 °C and progresses with maxima at around 360, 440 and 520 °C. These primary results indicate that carnauba wax is an attractive material to be used as carrier for flavours and aromas, especially those which are in liquid forms, since wax-aroma forms are easy to handle. We produced forms that can be used as food additives in animal feeds, while for human usage further study is needed in order to downscale particles and achieve desirable aroma release kinetics. For an increase of production scales, a careful selection of a suitable technique must be done. One possibility is to use the spray cooling technique because with this technique is possible to achieve high yields, and it can be run in both continuous and batch processing modes. There are other alternatives like the spinning disc technique, as an example of a solvent free process. For small scale productions, the technique of emulsification described here may be suitable as well.
